# Prediction of transcriptional regulatory elements for plant hormone responses based on microarray data

**DOI:** 10.1186/1471-2229-11-39

**Published:** 2011-02-24

**Authors:** Yoshiharu Y Yamamoto, Yohei Yoshioka, Mitsuro Hyakumachi, Kyonoshin Maruyama, Kazuko Yamaguchi-Shinozaki, Mutsutomo Tokizawa, Hiroyuki Koyama

**Affiliations:** 1 Faculty of Applied Biological Sciences, Gifu University, Yanagido 1-1, Gifu City, Gifu 501-1193, Japan; 2 Japan International Research Center for Agricultural Sciences, Ohwashi 1-1, Tsukuba, Ibaraki 305-8686, Japan

## Abstract

**Background:**

Phytohormones organize plant development and environmental adaptation through cell-to-cell signal transduction, and their action involves transcriptional activation. Recent international efforts to establish and maintain public databases of *Arabidopsis *microarray data have enabled the utilization of this data in the analysis of various phytohormone responses, providing genome-wide identification of promoters targeted by phytohormones.

**Results:**

We utilized such microarray data for prediction of *cis*-regulatory elements with an octamer-based approach. Our test prediction of a drought-responsive RD29A promoter with the aid of microarray data for response to drought, ABA and overexpression of DREB1A, a key regulator of cold and drought response, provided reasonable results that fit with the experimentally identified regulatory elements. With this succession, we expanded the prediction to various phytohormone responses, including those for abscisic acid, auxin, cytokinin, ethylene, brassinosteroid, jasmonic acid, and salicylic acid, as well as for hydrogen peroxide, drought and DREB1A overexpression. Totally 622 promoters that are activated by phytohormones were subjected to the prediction. In addition, we have assigned putative functions to 53 octamers of the Regulatory Element Group (REG) that have been extracted as position-dependent *cis*-regulatory elements with the aid of their feature of preferential appearance in the promoter region.

**Conclusions:**

Our prediction of *Arabidopsis cis*-regulatory elements for phytohormone responses provides guidance for experimental analysis of promoters to reveal the basis of the transcriptional network of phytohormone responses.

## Background

Phytohormones control plant morphology, development, and environmental adaptation through cell-to-cell signal transduction. They function not only independent as solo, but also in cooperative or competitive, interdependent ways in duos or trios. Altering the balance between auxin and cytokinin changes the fate of tissue differentiation *in vitro *[1]. Gibberellin has an antagonistic effect to abscisic acid for seed maturation and germination [2]. Ethylene activates auxin action by stimulation auxin biosynthesis and modulating auxin transport [3], and salicylic acid and jasmonic acid act competitively in pathogen responses [4]. A recent report suggests sequential activation of jasmonic acid, auxin, salicylic acid responses in mediating systemic acquired resistance [5]. These relationships between phytohormones are a part of the huge transcriptional network for complex phytohormone responses. Because of the biological importance of this network, intensive efforts have been dedicated for decades to the molecular identification of phytohormone receptors, transporters, intracellular signal transducers, transcription factors, and target promoters. Having gained understanding of several examples from hormone perception to gene activation, one of the most important current topics is how we understand the hormonal regulation of gene expression at the genome level, or the entire transcriptional network where multiple hormone responses intersect. Genome-wide determination of all the corresponding *cis*-regulatory elements is one of the challenges we should take up.

Previously, we have identified hundreds of promoter constituents by the LDSS (Local Distribution of Short Sequences) strategy, that is an *in silico *method to detect position-sensitive promoter elements regardless of their biochemical or biological roles [6,7]. Application of this method to the *Arabidopsis *genome resulted in the successful detection of 308 octamers that belong to a group of putative *cis*-regulatory elements, the Regulatory Element Group (REG), in addition to novel core promoter elements [8].

Comparison between the REG and reported *cis*-regulatory elements of *Arabidopsis *suggested that the elements identified in the REG include about half of the known *cis*-elements, the other half remaining undetected. These results, demonstrating the limited sensitivity of LDSS, were considered reasonable because LDSS has a methodological limitation in that it fails to detect *cis*-elements of the position-insensitive type [7,9].

The functions of half of the detected REGs remain unknown, and of the half known, their precise biological roles are not clear to date. In order to give biological annotation to REGs, we decided to utilize microarray data to predict the biological responses of *cis*-elements that are defined by the corresponding microarray experiments. Although there are several well-established methodologies for the prediction in motif-based search algorithms (Gibbs Sampler [10,11], MEME [11,12], and their parallel analysis platform, MELINA II [13]), we needed an octamer-based approach in order to give compatibility to REG analysis. In this report, we describe the development of an octamer-based prediction method using microarray data of phytohormone responses and all the predicted data by analysis of 622 hormone-responsive *Arabidopsis *promoters.

## Results

### Searching for overrepresented regions in a promoter with the aid of RAR

Our method is achieved in the following two steps. Firstly, the Relative Appearance Ratio (RAR) is calculated for each octamer (see methods). This comparative value indicates the degree of overrepresentation in a stimulus-responsive promoter set over a set of total genic promoters in a genome. A high RAR indicates enrichment of a corresponding octamer in the responsive promoter set, and thus octamers with high RARs are suggested to be involved in gene regulation that reflects the characteristics of the selected promoter set. Secondly, a prepared RAR table for all the octamers is applied to a specific promoter. This application is achieved by scanning the promoter with octamers giving the corresponding RAR values one by one.

### Scan of the drought responsive RD29A promoter

The RD29A promoter is one of the most characterized drought-responsive promoters having undergone intensive functional analyses, and several *cis*-regulatory elements in the promoter have been experimentally identified [14,15]. We applied our prediction method to the RD29A promoter to estimate the sensitivity and reliability of the prediction.

**Figure 1:**
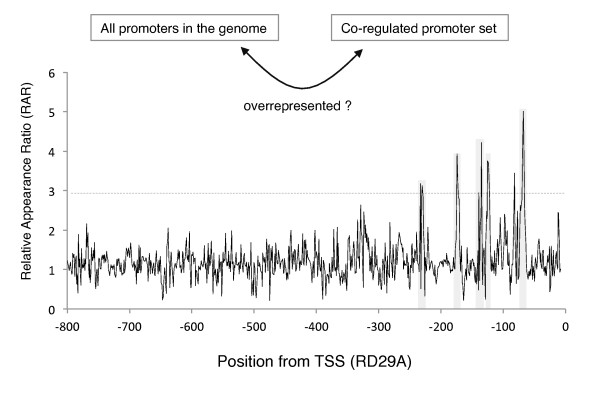
**Scanning of a promoter by a RAR table**. The Relative Appearance Ratio (RAR) that reflects the degree of overrepresentation in a selected set of 362 up-regulated promoters over the total promoters in a genome, is prepared for all the octamers, and the RAR table was applied to a drought-responsive promoter, RD29A. The promoter scanning was achieved by evaluation of octamers in the promoter sequence by 1 bp-steps. Horizontal dotted line shows a height of 3.0.

The results of promoter scanning of RD29A with a RAR table prepared with microarray data of drought treatment [16] are shown in Figure 1. The scan revealed several high RAR peaks between -300 to -50 relative to the transcription start site (TSS) (shaded area, Figure 1). These peaks predict *cis*-regulatory elements for drought response.

During the analysis of RD29A and others, we found that octamers with very high RAR values (20~100) are often very rare sequences among all the genic promoters (data not shown). One possible reason for these high values is statistical fluctuation. In order to avoid these potential false positives, we calculated *P *values for each octamer-RAR combination under the assumption of random distribution, and RAR with *P *> 0.05 was masked as zero. The resultant filtered RAR is referred to as RARf. As expected, a decrease in the number of octamers with a positive RAR (> 3) was observed only for fractions of rare octamers (Figure S1, Additional file 1).

**Figure 2:**
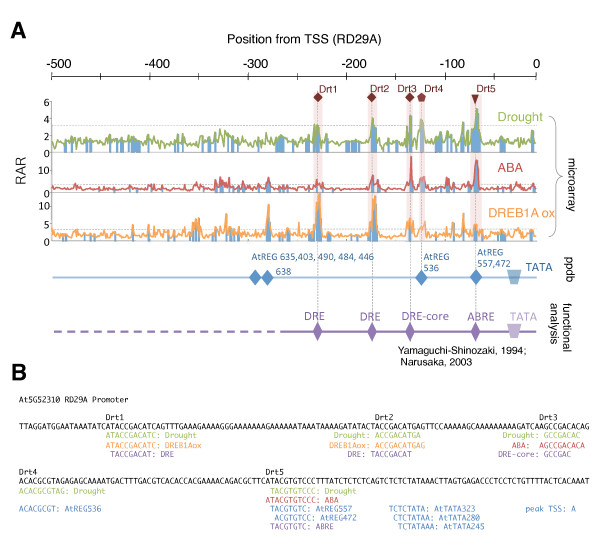
**Analysis of the RD29A promoter**. Panel A. The three graphs show scanning results based on microarray data of the drought response (green), the ABA response (red), and DREB1A overexpressors (orange). The regions filled with the blue bar indicate the statistically confident (*P *< 0.05) areas. Predicted *cis*-elements that are related to drought, ABA, and DREB1Aox are indicated as Drt1 to 5 (at top of the graphs). Blue line in the middle summarizes the prediction data by the ppdb, and elements in the REG in the promoter are shown. Purple line at the bottom shows *cis*-regulatory elements identified by functional analysis. Panel B. The sequence of RD29A promoter. Green, red and orange: predicted *cis*-elements from promoter scanning; blue: ppdb information; purple: functionally identified *cis*-elements.

Using the RARf, the RD29A promoter was scanned again (Figure 2). Panel A shows three independent information, that are summary of our predictions ("microarray" in the panel), information from Plant Promoter Database (ppdb), and functional analysis.

The top assembled graphs show scan data with the RAR and RARf tables for response to drought [16], response to ABA [17], and response to overexpression of DREB1A, a key transcription factor for cold and drought responses, in transgenic plants [18]. Lines show the RAR values for each promoter while filled (blue) bars indicate RARf values. Therefore, the open areas in the graphs are statistically insignificant whatever the RAR values are. According to the scan data, 5 sites, designated as Drt1 to 5, were selected as potential *cis*-regulatory elements for the drought response of RD29A. By comparing the peak heights of drought, ABA, and DREB1Aox, Drt1 and 2 are suggested to be sites for DREB1A-related drought response, Drt3 and 5 for ABA-mediated drought response, and Drt4 for drought response not mediated by DREB1A or ABA.

The second blue line shows information form the ppdb [19], and the database identify positions of REGs and a TATA box in the promoter. Of the identified REGs in the promoter, Drt4 and 5 coincide with AtREG536 and AtREG557/472, respectively. The predicted *cis*-elements at the sequence level are shown in Panel B. The rest Drt elements (1 to 3) do not have corresponding REGs.

The bottom purple line in the panel summarizes the results of functional analysis reported by Yamaguchi-Shinozaki *et al. *[14,15], and Narusaka *et al*. [15]. They have identified four *cis*-regulatory elements, DRE, DRE-core, and ABRE for the drought response, in addition to AS1 (not shown) that is a functional element not involved in the drought response.

Comparison of our predicted *cis*-elements (Drt1 to 5) with those already reported revealed reasonable results for our prediction as follows: 1) Drt1 and Drt2 are the site of a drought-responsive element, DRE [14,15], and include direct binding sequences of DREB1/2 [20,21], 2) Drt3 is a drought-responsive element [15] that has less conserved recognition sequence for DREB1/2 than Drt1/2 [21] and 3) Drt5 is an ABA-mediated drought responsive element, ABRE [15]. In addition, less direct reported evidence suggest as follows: 4) ABA-mediated activation of CBF4/DREB1D by drought stress [22] does support the idea ABA-mediated activation of RD29A *via *DRE-containing Drt3, 5) Drt4 partially matches with the barley Coupling Element 3 (CE3: AACGCGTGCCTC, underline sequence corresponds to Drt4) that cooperatively functions in ABA response with ABRE [23], suggesting a possible role of Drt4 in mediating ABA response. Although a motif for CE3, prepared from barley, maize, and rice promoters, is reported to be practically absent from the *Arabidopsis *genome [24], identification of a putative CE3 element from a drought-responsive promoter may suggest that *Arabidopsis *also uses CE3 with a different sequence preference from monocots.

In summary, our *cis*-element prediction of the RD29A promoter is good and there is no obvious conflict with functional studies. These results demonstrate that the methodology utilized provides prediction data that can support large-scale functional analysis at a practical confidence level.

### Two possible cases for cis-elements as indirect targets

**Figure 3:**
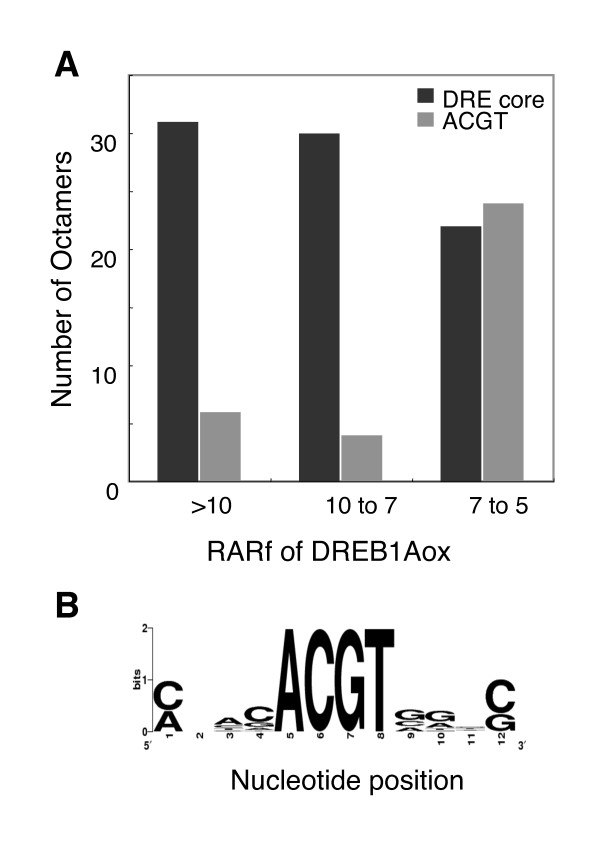
**DRE and ABRE detected by DREB1Aox**. Among the high RARf octamers for DREB1Aox, ones containing the DRE and ACGT (ABRE) motifs were selected, and the number of the octamers is shown according to their RARf values (**A**). DRE is the direct target of DREB1A, and ABRE is not. Selected octamers containing ACGT motif were aligned with ClustalW [37] and subjected to WebLogo [38] (**B**).

When we were preparing the RARf table for DREB1Aox, we found many ABRE-related sequences were present in the high RARf group, in addition to the expected DRE. For example, Table 1 shows REGs that have high RARf values of DREB1Aox. The highest REG has a DRE motif, but the lower ones in the table often contain the ACGT motif, that includes ABRE. Figure 3 shows the number of octamers that have a high RARf of DREB1Aox, and the figure also shows that both DREs and ACGTs are found in the high RARf group, and that DREs are higher than ACGTs.

**Table 1:** REGs with high RARf of DREB1Aox

REG ID	Octamer	Motif	DREB1Aox	ABA	Drought
AtREG638	AGTCGGTC	DRE	9.44	5.57	0
AtREG448	ATGCCACG		4.89	3.54	1.78
AtREG453	CACGTGTA	ACGT	4.81	5.47	2.36
AtREG557	GACACGTA	ACGT	4.66	8.19	3.00
AtREG472	ACGTGTCC	ACGT	4.60	11.95	3.24
AtREG478	ACGTGTCG	ACGT	4.41	10.48	5.77
AtREG489	ACGTCACG	ACGT	4.15	4.29	0
AtREG513	ACGTGGAC	ACGT	3.65	3.02	0
AtREG628	ACACGTGA	ACGT	3.64	2.67	1.90
AtREG428	ACGACACG		3.58	5.32	3.20
AtREG544	ACCACGTG	ACGT	3.51	4.35	2.48
AtREG612	GGCCCACA	GCCCA	3.33	0	0
AtREG527	AACGACAC		3.12	0	0
AtREG460	CACACGTG	ACGT	3.07	5.44	1.96

Calculation of the RARf is carried out in a direction-insensitive manner.

**Figure 4:**
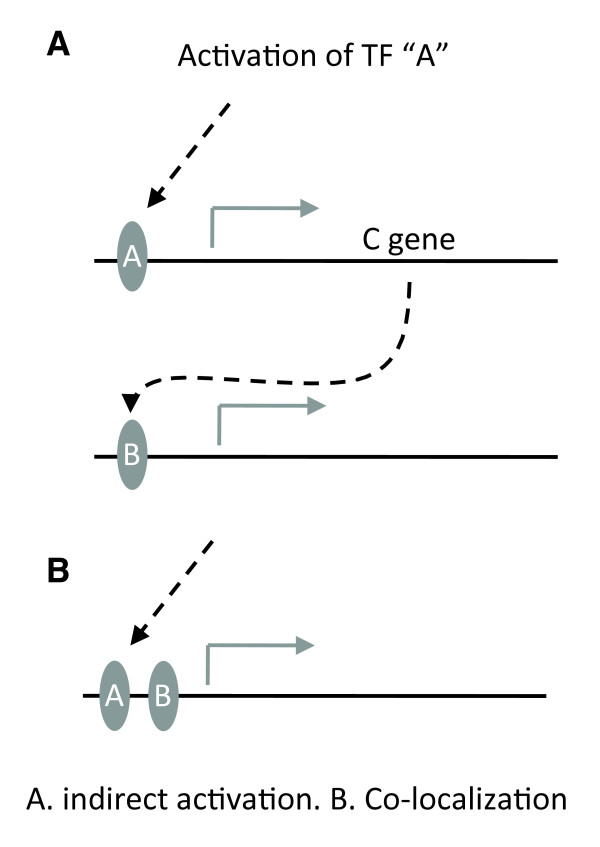
**Possible models for the selection of an indirect target**. For both panels, site A is the direct target of a transcription factor (TF) "A" and B is the indirect site. The figure illustrates two models for the detection of site B, in addition to site A. Panel A. Sequential model. One of the gene products activated by site A ('C gene' in the figure) targets site B. Panel B. Bystander model. Sites A and B coexist in the same promoter and may cooperatively function to activate the target promoter. Another possibility is that site B is not involved in the gene activation by TF "A" but is involved in a distinct signaling pathway, resulting in site A and B, having only a biological relationship. A possible example of this latter case is the coexistence of a site for an environmental response and for tissue-specific expression (e.g., light response and leaf-specific expression).

We put forward two hypotheses for the detection of ABRE (Figure 4). The first hypothesis is indirect stimulation of ABRE by DREB1A (Panel A). However, the ABA response is not suggested to be triggered by DREB1A [25], so this hypothesis is unlikely. The fact that there is no activation of *trans*-factors for ABRE, AREB1/2/ABF3 in DREB1A overexpressors [18] also opposes the hypothesis. The second hypothesis is the co-existence of DRE and ABRE in a same promoter. This can happen if these two motifs function cooperatively, or if there is no direct cooperation but they have a biological relationship that allows for independent DREB1A- and ABA- mediated signals on the promoter. In order to examine the second hypothesis, we looked at the possibility of the co-existence of RARf-positive DRE- and ACGT-related octamers. As shown in Table 2, these two groups do co-localize with each other. Therefore, the high RARf values of DREB1Aox for ABRE-related octamers are suggested to be a consequence of the second hypothesis (Panel B, Figure 4).

**Table 2:** Co-localization of DRE and ACGT elements with high RARfs of DREB1Aox

	All	ACGT	ACGT ratio
All	14960	2886	19.29%
DRE	2642	760	28.77%
DRE ratio	17.66%	26.33%	

The number of promoters is shown. The probability of this distribution based on Fisher's Exact Test is: *P *= 1.81E-17.

Figure 3B shows a sequence motif of the ACGT-containing octamers colocalizing with the DRE in the 760 promoters shown in Table 2. The motif has a bias toward ABRE (PyACGTGGC, [25]) as shown at the 9th (G) and 10th (G) positions.

### Cis-element prediction for phytohormone responses

Subsequently, we analyzed microarray data of phytohormone responses in shoots. The data source is listed in Table 3. Using the same methodology as for the analysis of the drought response, RAR and RARf tables were calculated for each microarray data, and then octamers with high RARf values (RARf > 3) were extracted. As shown in Table 3, 500 to 1,400 octamers, have been selected as having a high RARf for each phytohormone, and in total 7,983 octamers were picked-up. This large number might suggest the inclusion of false-positives in spite of the filtering. The number of REGs in the predicted sequences is 53 out of 308 in total, and the prediction for the REG octamer would not be as overestimated as for the non REG-type octamers. All the REGs identified in these analyses are shown in Table 4. These data will be incorporated to our promoter database, the ppdb [19] in the near future.

**Table 3:** Extraction of overrepresented octamers in promoters with hormone and drought responses

Microarray	Ref	Selected promoter	REG number^1^	Octamer number
ABA	TAIR_ME00333 [17]	98	40	1,370
Ethylene	TAIR_ME00334 [17]	88	1	1,162
BL	TAIR_ME00335 [17]	82	0	943
CK	TAIR_ME00356 [17]	165	4	1,105
Auxin	TAIR_ME00336 [17]	67	3	1,008
JA	TAIR_ME00337 [17]	254	2	577
SA	TAIR_ME00364 [17]	197	0	813
H_2_O_2_	[39]	260	7	614
Drought	TAIR_ME00338 [16]	362	14	559
DREB1A ox	MEXP-2175 [18]	81	23	1,106
any treatment			53	7,983
all			308	65,536

Data for responses in shoots or seedlings were selected. ABA: 10 uM abscisic acid for 1 h; ethylene: 10 uM ACC for 3 h; BL: 10 nM brassinolide for 3 h; CK: 1 uM zeatin for 3 h; auxin: 1 uM IAA for 3 h; JA: 10 uM methyl jasmonate for 3 h; SA: 10 uM salycilic acid for 3 h; H_2_O_2_: 3% solution for 3 h; drought: 1 h-treatment; DREB1Aox: constitutive overexpression of DREB1A driven by a 35S promoter. ^1^Count of complementary sequence is merged because REG is defined as orientation-insensitive.

**Table 4:** Identification of hormone-responsive REGs

REGs with high RARf values												
**REG ID**	**oct**	**ABA**	**Ethylene**	**BL**	**CK**	**Auxin**	**JA**	**SA**	**H_2_O_2_**	**Drought**	**DREB1Aox**	**annotation**

AtREG366	CACGTGTC	9.132	0	0	0.344	0	0	0	0.492	2.747	2.631	ABA
AtREG367	CACGTGGC	6.309	0	0	0.363	0	0	0	0	2.204	2.462	ABA
AtREG371	ACGTGGCG	6.427	0	0	0	0	0	0	0	2.066	0	ABA
AtREG379	ACGTGGCA	3.464	0	0	0	0	0	0	0	1.765	2.959	ABA
AtREG382	ACACGTGG	7.351	0	0	0	0	0	0	0	2.671	0	ABA
AtREG389	ACGTGTCA	5.964	0	0	0	0	0	0	0	2.069	2.255	ABA
AtREG404	CCCGGCCC	0	0	0	4.197	0	0	0	0	0	0	CK
AtREG408	CACGTGGA	6.095	0	0	0	0	0	0	0	2.406	0	ABA
AtREG428	ACGACACG	5.324	3.294	0	0	0	2.283	0	0	3.203	3.579	ABA, DREB1Aox, Ethylene, Drought
AtREG438	ATGACACG	3.409	0	0	0	0	0	0	0	0	0	ABA
AtREG440	CACGTCAG	4.46	0	0	0	0	0	0	0	0	0	ABA
AtREG441	AACCGCGT	0	0	0	0	0	0	0	2.6	3.969	0	Drought
AtREG446	ATTGGCCC	0	0	0	3.137	0	0	0	0	0	0	CK
AtREG448	ATGCCACG	3.538	0	0	0	0	0	0	0	1.779	4.892	ABA, DREB1Aox
AtREG450	ACGTGGCT	3.3	0	0	0	0	0	0	0	0	0	ABA
AtREG453	CACGTGTA	5.469	0	0	0	0	2.59	0	0	2.355	4.812	ABA, DREB1Aox
AtREG457	CCGGCCCA	0	0	0	4.458	0	0	0	0	0	0	CK
AtREG460	CACACGTG	5.438	0	0	0	0	0	0	0	1.963	3.07	ABA, DREB1Aox
AtREG464	CACGTGGG	3.333	0	0	0	3.9	3.086	0	0	0	0	ABA, Auxin, JA
AtREG466	CACGTCAC	3.689	0	0	0	0	0	0	0	0	0	ABA
AtREG468	CGTGGCAG	3.422	0	0	0	0	0	0	0	0	0	ABA
AtREG470	ACGTGTCT	5.361	0	0	0	0	0	0	0	1.964	0	ABA
AtREG471	CGTGGCGA	6.784	0	0	0	0	0	0	0	0	0	ABA
AtREG472	ACGTGTCC	11.95	0	0	0	0	0	0	0	3.235	4.6	ABA, DREB1Aox, Drought
AtREG478	ACGTGTCG	10.48	0	0	0	0	2.285	0	0	3.577	4.41	ABA, DREB1Aox, Drought
AtREG481	GACACGTC	5.088	0	0	0	0	0	0	0	0	0	ABA
AtREG488	CCGCGTTA	0	0	0	0	0	0	4.104	0	2.792	0	SA
AtREG489	ACGTCACG	4.287	0	0	0	0	0	0	0	0	4.15	ABA, DREB1Aox
AtREG498	CGTGTCAC	4.889	0	0	0	0	0	0	0.205	2.059	0	ABA
AtREG502	CCGCGTGA	0	0	0	0	0	0	0	0	3.834	0	Drought
AtREG513	ACGTGGAC	3.018	0	0	0	0	0	0	0	0	3.652	ABA, DREB1Aox
AtREG515	ACGTCAGC	2.858	0	0	0	0	0	3.413	0	0	0	SA
AtREG517	ACACGTCA	5.332	0	0	0	0	0	0	0	0	0	ABA
AtREG527	AACGACAC	0	0	0	0	0	0	0	0	0	3.122	DREB1Aox
AtREG536	ACACGCGT	6.784	0	0	0	0	0	0	0	3.214	0	ABA, Drought
AtREG544	ACCACGTG	4.347	0	0	0	0	0	0	0	2.484	3.506	ABA, DREB1Aox
AtREG547	ACGTGGAT	3.101	0	0	0	0	0	0	0	1.679	0	ABA
AtREG553	CAACGGTC	0	0	0	0	5.769	0	0	0	0	0	Auxin
AtREG557	GACACGTA	8.185	0	2.877	0	0	0	0	0	2.998	4.66	ABA, DREB1Aox
AtREG560	CCGCCACG	4.988	0	0	0	0	0	0	0	0	0	ABA
AtREG562	ACGTGTAC	4.064	0	0	0	3.303	0	0	0	1.956	0	IN tabl
AtREG578	ACGTCATC	3.34	0	0	0	0	0	1.994	0	0	0	ABA
AtREG588	ACGTGTGA	3	0	0	0	0	0	0	0	0	2.722	ABA
AtREG590	AACACGTG	7.004	0	0	0	0	3.541	0.36	0	2.942	0	ABA, JA
AtREG595	ACCCCTGA	0	0	0	3.817	0	0	0	0	0	0	CK
AtREG606	ACGTGACA	3.205	0	0	0	0	1.855	2.391	0	0	0	ABA
AtREG608	AAGCCACG	3.053	0	0	0	0	0	0	0	0	0	ABA
AtREG612	GGCCCACA	0	0	0	2.858	0	0	0	0	0	3.327	DREB1Aox
AtREG615	GGGACCCA	4.26	0	0	0	0	0	0	0	0	0	ABA
AtREG628	ACACGTGA	2.672	0	0	0	0	2.835	0	1.888	1.899	3.637	DREB1Aox
AtREG631	CGCGTGAA	0	0	0	0	0	0	0	0	3.332	0	Drought
AtREG638	AGTCGGTC	5.571	0	0	0	0	0	2.771	0	0	9.436	DREB1Aox, ABA
AtREG646	CGTAATTA	3.016	0	0	0	0	0	0	0	0	0	ABA

Data of the complementary sequence is merged.

### Evaluation of prediction

The prepared RARf tables for various hormone responses enable *cis*-element predictions of hormone-responsive promoters. Our prediction based on the RARf tables was then evaluated with the aid of published results. Articles were surveyed reporting identification of *cis*-elements for hormone or drought responses of *Arabidopsis *promoters. During the search, we noticed that most of the previous articles analyzing phytohormone-responsive promoters have an objective of finding *at least *one *cis*-element that enables the responses, and only a few article tried to identify *all *the regulatory elements within a promoter of interest. We selected a few articles analyzing RD29B and PR1 promoters, in addition to ones dealing with RD29A as we have seen before. These articles include systematic linker scan analysis or intensive functional analysis.

Subsequently, we did promoter scan using appropriate RARf tables (drought for RD29B and SA for PR1), and peaks with a height over 3.0 were selected as predicted *cis*-elements. Table 5 shows comparison of predicted and experimentally confirmed *cis*-elements detected from the intensively analyzed regions of the three promoters. As shown in the table, majority of the prediction fit with the experimental results ("Positive" in the Prediction assessment column). "False positive" in the column means these loci are predicted as *cis*-elements but have conflicts with reported experimental results. Besides real failure of prediction, we suggest two possible reasons for the disagreement. One is difference between physiological (and experimental) conditions for preparation of RARf tables and reported promoter analyses. Another possible reason is related to sensitivity of detection of transcriptional responses. For example, -669 of the PR1 promoter (Table 5) was concluded as no contribution to the salicylic acid response using the GUS reporter (LS5) [26], but utilization of more sensitive LUC reporter could detect SA-response by LS5 [27]. This example demonstrate importance of selection of reporter genes for assays, and documents the reported promoter analysis may provide rather tentative results. These possible reasons lead underestimation of the assessment shown in Table 5.

**Table 5:** Verification of prediction by experimental analysis

AGI code	Position from TSS^1^	RARf		Predicted *cis*-element	REG	Prediction assessment	Reference^4^	Response	Element name	MEME	Gibbs Sampler
		Drought^2^	SA^3^								
AT5G52310 (RD29A)	-231	3.12		ATACCGACATCA		**Positive**	Yamaguchi-Shinozaki, 1994	Drought	DRE	No detect.	No detect.
	-175	3.94		ACTACCGACATGAG		**Positive**	Narusaka, 2003	Drought	DRE	No detect.	No detect.
	-137	4.22		AAGCCGACACA		**Positive**	Narusaka, 2003	Drought	DRE-core	No detect.	No detect.
	-125	3.76		ACACGCGTAGA	AtREG536	?^5^	Narusaka, 2003	Drought		No detect. ^7^	No detect.
	-82	3.44		ACAGACGC		**False positive**	Yamaguchi-Shinozaki, 1994	Drought		No detect	No detect
	-71	5.01		ATACGTGTCCCT	AtREG557,472	**Positive**	Narusaka, 2003	Drought	ABRE	No detect.	No detect.^7^
	
AT5G52300 (RD29B)	-163	3.16		CGTACGTGTCA	AtREG450	**False positive**	Uno, 2000	Drought		No detect	No detect^7^
	-137	*				**Absent^7^**	Uno, 2000	Drought	ABRE	No detect.	No detect.^7^
	-112	3.21		GTACGTGTCA	AtREG557, 389	**Positive**	Uno, 2000	Drought	ABRE	No detect.	No detect. ^7^
	
AT2G14610 (PR1)	-669		3.82	ACGTCACT		**Positive**	Pape, 2010	INA^6^/SA	LS5	No detect.	No detect.
	-657		6.38	TACTTACGTCAT		**Positive**	Lebel, 1998; Pape, 2010	INA^6^/SA	LS7	No detect.	No detect.
	-607		3.65	TAGGCAAG		**False positive**	Lebel, 1998	INA^6^/SA		No detect	No detect

^1^Position from major TSS data from ppdb. ^2^1 h-treatment. ^3^See Table 3 for experimental conditions. ^4^Source of functional analysis. *RARf for ABA response is 3.7. ^5^Lack of the corresponding functional data. ^6^INA: 2,6-dichloro isonicotinic acid, a SA analog. ^7^Detected with the promoter set of ABA response. For analysis of RD29B by MEME and Gibbs Sampler, it was included to the applied promoter set. Promoter scan for prediction was achieved for the regions where linker scan or intensive functional analyses were achieved, and peaks with RARf > 3.0 were selected as prediction. Utilized RARf tables are shown in the table.

For comparison, motif extraction by MEME and Gibbs Sampler was achieved using the same promoter sets used to prepare the RARf tables. As shown in the left two columns, promoter sets of drought and SA responses failed to detect any motifs in RD29A/B and PR1 promoters, respectively. Further analysis showed the promoter set of ABA response could detect some of the *cis*-elements in RD29A and RD29B promoters. These comparisons revealed considerably higher sensitivity of the RARf-based approach than conventional MEME and Gibbs Sampler.

Results shown in Table 5 are summarized in Table 6. The table shows efficient success rate (58 ~ 67%) and high sensitivity (Cover rate, 88 ~ 89%). These results demonstrate our prediction based on the prepared RARf tables are well effective, and useful as a guide for experimental promoter analysis.

**Table 6:** Summary of prediction assessment

Method	Prediction	Positive	False positive	Absent	Success rate	Cover rate
RARf-based scan	12	7	3	1	58~67%	88~89%
MEME	0	0	0	9	0%	0%
Gibbs Sampler	0	0	0	9	0%	0%

Results of Table 5 are summarized.

**Figure 5:**
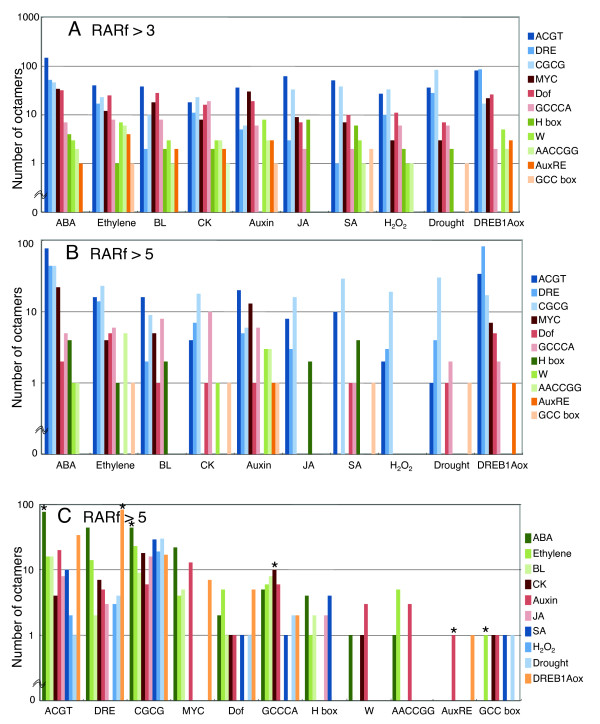
**Recognition motifs by transcription factors of high RARf octamers**. The number of high RARf octamers is shown in regard to sequence motifs. A. Octamers with RARf values of more than 3 are shown according to phytohormone responses. B. Octamers with RARf values of more than 5 are shown according to phytohormone responses. C. Octamers with RARf values of more than 5 are shown according to sequence motifs. Data marked with asterisks are mentioned in the text.

We then checked if the high RARf octamers contained the sequences expected. Table 7 shows a list of transcription factor-recognition sequences. According to our current knowledge, the ABA response is in part mediated by ABRE, an ACGT-related motif, the auxin response by AuxRE, and the ethylene response by the GCC box. Classification of high RARf octamers by these motifs revealed complex results (Figure 5A). This complexity is due in part to the intricate nature of the transcription network, and also to the detection of indirect *cis*-elements.

**Table 7:** List of transcription factor-recognition motifs

Motif name	Transcription factors	Motif	Response	Reference
ACGT	bZIP, PIF, bHLH	ACGT	ABA (ABRE), various environmental stimuli including light (G box) and biotic stress (G box)	[40]
DRE	DREB1/2 (ERF/AP2 subfamily)	CCGAC	Cold, drought	[25]
CGCG	AtSR	CGCG^1^	Various stresses	[41]
Myc	Myc	CANNTG	ABA	[42]
Dof	Dof	AAAG	Various regulation	[43]
GCCCA	TCP	GCCCA	Meristematic expression	[6]
H box	MYB	CCTACC	Biotic stress	[44]
W box	WRKY	TTGAC(C/T)	Biotic stress, ABA, senescence	[45]
AACCGG	unknown	AACCGG		[6]
AuxRE	ARF	TGTCTC	Auxin	[46]
GCC box	ERF/AP2	AGCC(A/G)CC	Ethylene, biotic stress	[44]

^1^Defined in this study.

Elevation of the cut-off value for the RARf from 3 to 5 resulted in a reduction in octamer numbers, and a change in distributions along motifs, resulting in clearer characteristics for each group of response (Panel B). Panel B shows the result as follows: the most major octamers for the ABA response have the ACGT motif, and the ones for DREB1Aox have DRE. The most major octamers for ethylene and auxin were expected to be the GCC box and AuxRE, respectively, but this was not the case. One possible reason for this is the difference in stringency for each motif. For example, ACGT and CGCG are tetramers, but AuxRE and the GCC box are defined as heptamers, so comparison of octamer numbers with these motifs is not fair. In order to overcome such inequalities, high RARf octamers were re-organized according to each motif (Panel C). The panel shows that the highest octamer number for ACGT comes from ABA, and DRE from DREB1Aox, again giving reasonable results. The number of octamers for AuxRE and the GCC box groups is much fewer than for the groups of ACGT or DRE, as expected. The highest numbers for AuxRE and the GCC box come from treatments including auxin and ethylene, respectively. GCCCA, an element for cell proliferation-dependent expression [6], contains CK (cytokinin) as the most major response group. All these results (asterisked in Panel C) revealed our prediction is good, and agrees with our current knowledge on transcriptional responses to phytohormones.

Preparation of reliable RARf tables allows us to scan native promoters. We next scanned 622 promoters that showed 5-fold or more activation by phytohormones with the corresponding RARf tables. The combination of the scanned promoters and applied RARf tables is shown in Table S1 (Additional file 2), and all the high RARf regions (> 3) of the analyzed promoters are shown in Table S2 (Additional file 3). The table also gives information of the corresponding positions, sequences, REG IDs, and also the presence of transcription factor-recognition motifs listed in Table 7. The prediction data for the 622 hormone-activated promoters helps functional analysis of individual promoters, and also evaluation of sequence polymorphism among accessions in these promoters.

### Possible crosstalk

There are two types of signaling crosstalk that can be observed in the promoter region: 1) merging of two distinct signals on a *cis*-element, and 2) merging of two signals on a promoter by the co-existence of corresponding *cis-*elements. In this report, we provide information for the former situation by analyzing native promoters that show hormone responses.

**Figure 6:**
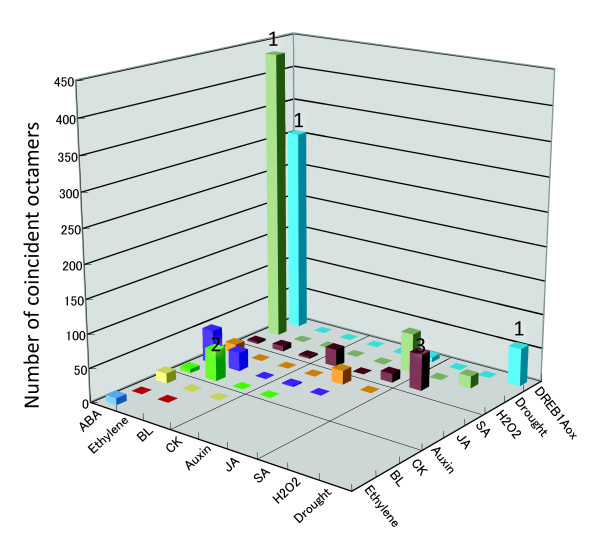
**Possible crosstalk at predicted *cis*-elements**. The number of octamers that were coincidently detected by two phytohormone responses is shown. When the distance of two octamers is 4 pb or less, they were counted as having coincident localization. The numbers at the top of bars (1 to 3) indicate the following crosstalk, and are mentioned in the text. 1: ABA ~ Drought ~ DREB1Aox, 2: Ethylene ~ Auxin, 3: SA ~ H_2_O_2_.

From the scanned data of 622 native promoters, we extracted overlapping octamers with high RARf values for multiple RARf tables. Table S3 (Additional file 4) shows all the overlapping high RARf octamers whose distance is 4 bp or less. The obtained data was summarized in Figure 6. From the data, we suggest three examples of predicted crosstalk as indicated in the graph. 1) ABA ~ Drought ~ DREB1Aox. This crosstalk is biologically reasonable, as we have seen during the analysis of the RD29A promoter. 2) Ethylene ~ Auxin. In agreement with the predicted crosstalk, two types of regulation of the auxin response by ethylene are known. One is activation of auxin biosynthesis by ethylene [3,28], and the other is elevation of auxin concentration by modulation of auxin transport by ethylene [3,29]. 3) SA ~ H_2_O_2_. SA-induction of H_2_O_2 _accumulation is reported [30]. Again, these analyses suggest the prediction of *cis*-elements is reliable.

### Framework for cis-element prediction

**Figure 7:**
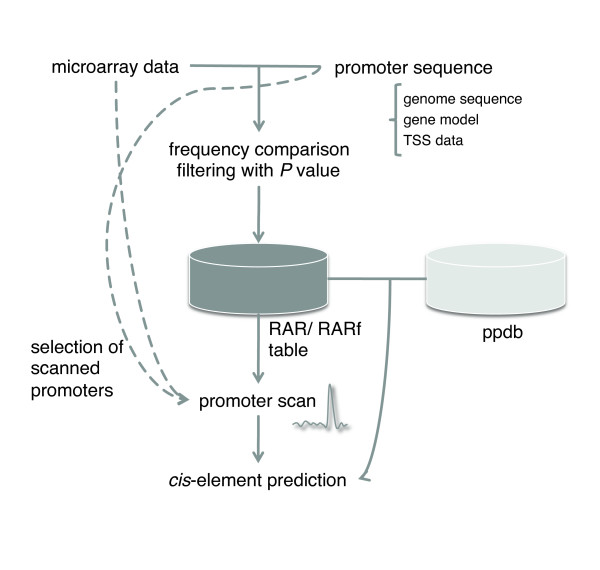
**Data flow of our prediction**. The data sources of the analysis are microarray data, promoter sequence, and ppdb data based on LDSS analysis. The possible outputs of the analysis are a list of high RARf octamers, promoter scan data, and a list of high RARf regions in the scan data.

Figure 7 illustrates a framework for *cis*-element prediction developed in this study. As shown, microarray data and promoter sequence are used for the promoter scan. The REG and also the sequence of core promoter elements are derived from the ppdb, and this information is added to high RARf octamers. The promoter scan data is the final output of the analysis.

## Discussion

Confirmation of our established prediction scheme, although not a novel methodology, has revealed that the output prediction data is reasonable and acceptable as a working hypothesis for experimental verification. Our predictions have been shown to include indirect targets in addition to direct ones (Figure 3, 4, and Table 2), but this problem can be handled more easily if users are aware of it. One possible approach to avoid indirect targets might be by the utilization of a more stringent threshold for RARf. However, we suggest that this approach is not practical because the population of high RARf octamers varies considerably according to the microarray experiment. For example, while many DRE-containing octamers have RARf values of DREB1Aox between 10 and 5, there are few octamers in such a range for drought response. We suggest that this variation in octamer population reflects the physiological complexity of the response. According to this idea, the drought response is more complex and diverse than that of to DREB1A overexpression. In short, fine-tuning of the cutoff value for RARf values should be done for each RARf table, and thus is not an easy approach. Our solution is to set a rather loose threshold (RARf > 3) and then for users to carefully interpret the prediction. This strategy can keep high sensitivity.

MEME and Gibbs Sampler are popular extraction methods of motifs that appear in an input sequence set. Because they are not good at detection of minor motifs in the input population, preparation of precise (not too large) size of the input where majority of the population have the target motifs is critical for successful extraction. In this point of view, it would be reasonable that they could detect some of the motifs in RD29A/B promoters using the ABA-responsive set but failed using the drought-responsive one, because drought stress would activate much more dispersed signaling pathways than ABA application. Remarkably, our RARf-based prediction could detect *cis*-elements using the drought-responsive set with high sensitivity (88 ~ 89%), demonstrating superiority of the RARf-based comparative approach in sensitivity and thus utility.

While promoter scanning with RARf tables is a straightforward way for the analysis of specific promoters of interest, there is a benefit. The scanning method can reduce false-positive sequences in the RARf tables, because octamers that do not exist in the analyzed promoters are neglected. In this article, we set a differential selection of promoters for the preparation of the RARf tables (> 3 fold activation in gene expression) and for scanned promoter sets (> 5 fold). This differential selection is a strategy to remove some of the false-positive octamers.

As a huge collection of plant microarray data (ArrayExpress) has been established, our analysis scheme, shown in Figure 7, allows us to predict *cis*-elements not just for hormone responses. Although functional validation of predicted *cis*-elements needs to be done by specialized plant physiologists in each research field, the prediction itself can be done by non-specialists, allowing extensive prediction that can support wide aspects of plant physiological studies.

In order to prove the biological roles of the predicted *cis*-elements, the elements need to be subjected to experimental verification. This can be achieved in two ways: loss-of-function experiments by introducing point mutations into the target promoters, and gain-of-function experiments using a synthetic promoter approach. The experimental methodologies for both approaches have been well paved, so there will be no technical problems in the verification. Our prediction data for phytohormone responses is therefore expected to be utilized for such experimental analyses. In our preliminary experiments for the identification of *cis*-elements for toxic aluminum ion responses in roots, accuracy of our *de novo* prediction is suggested to be high, just as in the case of the RD29A promoter (Kobayashi Y, Yamamoto YY, and Koyama H, unpublished results).

RD29A is one of the most intensively analyzed promoters whose function has been studied for more than a decade [25]. Therefore, we were surprised to find a novel putative *cis*-element (Drt4) that has not been noticed in previous experimental analyses. These findings may suggest that with the established promoter analysis, even if it is intensively done, there is the possibility that functional elements may be overlooked. This idea should not be surprising, because traditional promoter analysis (5' deletions, gain-of-function-experiments by core promoter swaps and point mutations) is designed to identify *at least one *functional elementfor the expected biological response, and not to determine the entire promoter structure. In order to understand the entire promoter structure, we suggest that bioinformatics-guided analysis is now indispensable.

## Conclusions

In this study, we utilized *Arabidopsis *microarray data to predict *cis*-regulatory elements for ABA, auxin, brassinolide, cytokinin, ethylene, jasmonic acid, salicylic acid, and hydrogen peroxide, in addition to drought response and DREB1A-mediated gene activation, from total 622 responsive promoters. These results provide opportunities to analyze promoter function by prediction-oriented approaches. Microarray data is also utilized to give annotation of REGs, that have been predicted as *cis*-regulatory elements dependent of promoter position in our previous analysis. The annotated REGs will be used in ppdb, Plant Promoter Database.

## Methods

### Promoter sequence

Promoter sequences from -1,000 to -1 relative to the major TSS were prepared for 14,960 *Arabidopsis *genes. The major TSS was determined by large scale TSS tag sequencing [8] or 5' end information of RAFL cDNA clones [19,31]. The Arabidopsis genome sequence and its gene models were obtained from TAIR [32].

### Preparation of RAR tables and promoter scanning

Microarray data (Table 3) was used to prepare lists of genes that showed expression of more than 3.0 fold above the control. Treatments that gave high RAR values with lower *P *values were selected. The RAR for each octamer was calculated from the following formula using home-made C^++ ^and Perl programs, and also Excel (Microsoft Japan, Tokyo).

RAR = (count in an activated promoter set/number of promoters in the set)/(count in total promoters/number of total promoters)

For each octamer-RAR combination, the *P *value was calculated by Fisher's Exact Test. The *P *values were transformed into LOD scores, and RAR values with a LOD score of less than 1.3 (*P *= 0.05) were filtered out to set as 0. The masked RAR values are referred to as RARf values in this report. RAR and RARf values for the REG annotation (Table 4) were calculated in a direction-insensitive manner, where information of the complementary octamer was merged.

Promoter scanning with RAR, RARf and LOD tables was achieved using homemade-Perl scripts and Excel. Promoters used for scanning showed over 5 fold-activation by hormone treatments. Cut-off value of RARf was set as 3.0 in order to pick up all the potential *cis*-elements, leaving the other sequences that are not worth further analysis. Because of this selection policy, secondary selection after promoter scanning is necessary for more reliable prediction. Threshold for the selection should be determined according to the utilized microarray experiments and also scanned promoters.

The same promoter sets used for preparation of RAR/RARf tables were applied to motif extraction by MEME and Gibbs Sampling methods at Melina II [13,33].

### Motif expression by WebLogo

Selected ACGT-containing octamers were aligned with ClustalW [34], considering counts of appearance, and subsequently subjected to WebLogo for the sequence logo expression as shown in Figure 3B[35].

### Data release

The promoters containing the REGs shown in Table 4 can be viewed at the ppdb (Plant Promoter Database, [19,36]). The REGs' annotation describing their possible roles (Table 4) will be incorporated into the ppdb in the near future. Raw scanning data of the 622 hormone-activated promoters will be supplied upon request.

## List of abbreviations

ABA: abscisic acid; ABRE: ABA responsive element; BL: brassinolide; CK: cytokinin; DRE: drought responsive element; INA: 2,6-dichloro isonicotinic acid; JA: jasmonic acid; RAR: relative appearance ratio; RARf: relative appearance ratio filtered; SA: salicylic acid; TSS: transcription start site.

## Authors' contributions

YYY designed and performed the analyses. YY and HM prepared public microarray data for calculation of RAR/RARf tables. KM and KYS prepared microarray data of DREB1Aox. MT and HK helped calculation of *P*-values for RARf preparation. All authors read and approved the final manuscript.

## Supplementary Material

Additional file 1 **Figure S1: Filtering of octamers by RARf**. Number of octamers showing high RAR values (> 3) is shown regarding total count of each octamers among 14,498 genic promoters. Rare octamers in the promoter region are shown to be filtered out by this statistical evaluation. Click here for file

Additional file 2 **Table S1: List of scanned promoters**. Combinations of promoters and RARf tables used for promoter scan are shown. Totally 622 promoters that show response to any phytohormones were selected for the scanning. All the detected signals are shown in Additional file 2 (Table S2). Click here for file

Additional file 3 **Table S2: Peaks of the scanned promoters**. All the peaks detected by 730 scanning data for the 622 promoters shown in Additional file 2 (Table S1) were extracted and shown. Position means distance from the major TSS used in ppdb. Corresponding REG ID and recognition motif are also indicated. Click here for file

Additional file 4 **Table S3: Possible cross-talk at regulatory elements**. Coincident detection by two different RARf tables is shown. If distance of two peaks by different RARf tables is within 4 bp, they are considered as co-localized and incorporated into the table. Totally 1188 co-localized peaks were detected. Position means distance from the major TSS used in ppdb. This table is the basis of Figure 6. Click here for file
