# Supplementary material for: Prediction of transcriptional regulatory elements for plant hormone responses based on microarray data
Source: BMC Plant Biol. 2011 Feb 24;11:39. doi: 10.1186/1471-2229-11-39 (PMC3058078; doi:10.1186/1471-2229-11-39)
Supplement: Additional file 1 — Figure S1: Filtering of octamers by RARf. Number of octamers showing high RAR values (> 3) is shown regarding total count of each octamers among 14,498 genic promoters. Rare octamers in the promoter region are shown to be filtered out by this statistical evaluation. [file 1471-2229-11-39-S1.PDF]

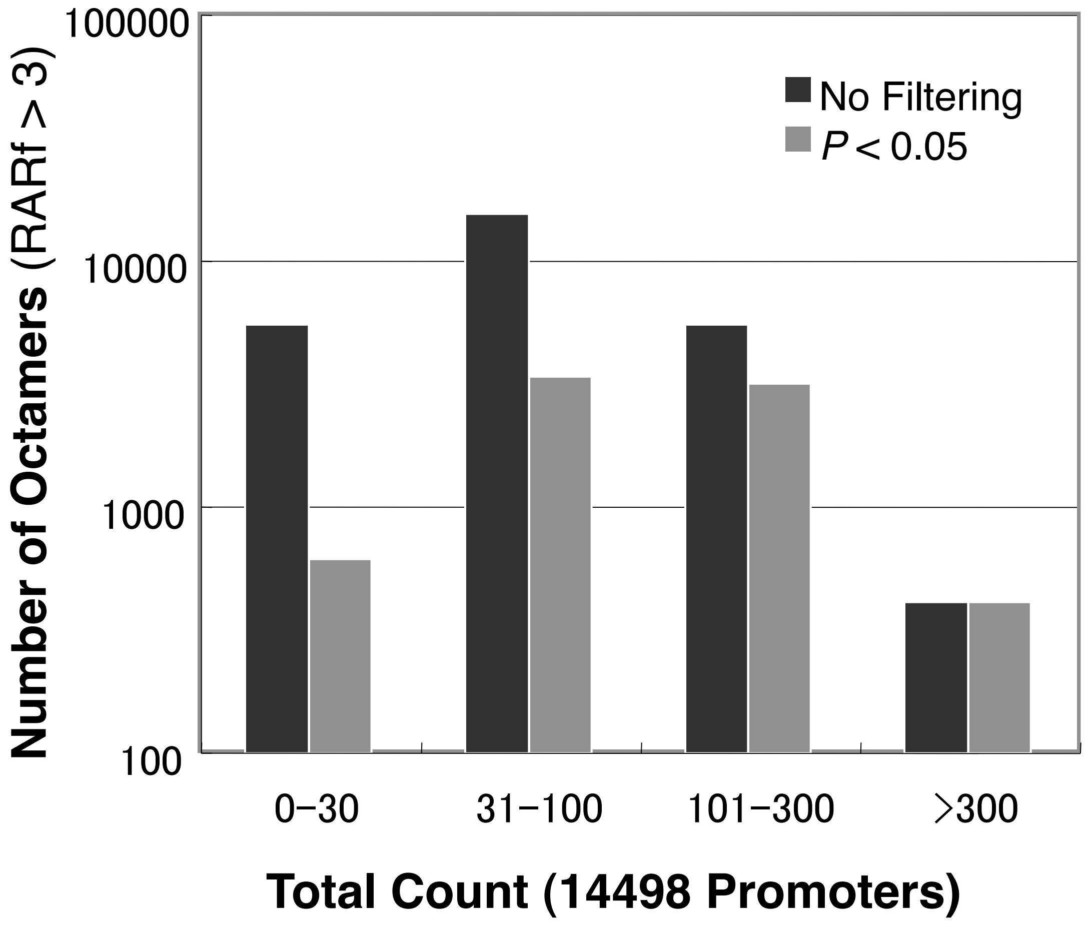

Figure S1. Filtering of octamers by RARf

Number of octamers showing high RAR values (>3) is shown regarding total count of each octamers among 14,498 genic promoters. Rare octamers in the promoter region are shown to be filtered out by this statistical evaluation.
